# Lightweight Payload Encryption-Based Authentication Scheme for Advanced Metering Infrastructure Sensor Networks

**DOI:** 10.3390/s22020534

**Published:** 2022-01-11

**Authors:** Nasr Abosata, Saba Al-Rubaye, Gokhan Inalhan

**Affiliations:** School of Aerospace, Transport and Manufacturing, Cranfield University, Cranfield MK43 0AL, UK; S.alrubaye@cranfield.ac.uk (S.A.-R.); Inalhan@cranfield.ac.uk (G.I.)

**Keywords:** sensors, security, IoT, CoAP, DTLS, AES, payload encryption, lightweight

## Abstract

The Internet of Things (IoT) connects billions of sensors to share and collect data at any time and place. The Advanced Metering Infrastructure (AMI) is one of the most important IoT applications. IoT supports AMI to collect data from smart sensors, analyse and measure abnormalities in the energy consumption pattern of sensors. However, two-way communication in distributed sensors is sensitive and tends towards security and privacy issues. Before deploying distributed sensors, data confidentiality and privacy and message authentication for sensor devices and control messages are the major security requirements. Several authentications and encryption protocols have been developed to provide confidentiality and integrity. However, many sensors in distributed systems, resource constraint smart sensors, and adaptability of IoT communication protocols in sensors necessitate designing an efficient and lightweight security authentication scheme. This paper proposes a Payload Encryption-based Optimisation Scheme for lightweight authentication (PEOS) on distributed sensors. The PEOS integrates and optimises important features of Datagram Transport Layer Security (DTLS) in Constrained Application Protocol (CoAP) architecture instead of implementing the DTLS in a separate channel. The proposed work designs a payload encryption scheme and an Optimised Advanced Encryption Standard (OP-AES). The PEOS modifies the DTLS handshaking and retransmission processes in PEOS using payload encryption and NACK messages, respectively. It also removes the duplicate features of the protocol version and sequence number without impacting the performance of CoAP. Moreover, the PEOS attempts to improve the CoAP over distributed sensors in the aspect of optimised AES operations, such as parallel execution of S-boxes in SubBytes and delayed Mixcolumns. The efficiency of PEOS authentication is evaluated on Conitki OS using the Cooja simulator for lightweight security and authentication. The proposed scheme attains better throughput while minimising the message size overhead by 9% and 23% than the existing payload-based mutual authentication PbMA and basic DTLS/CoAP scheme in random network topologies with less than 50 nodes.

## 1. Introduction

**Figure 1:**
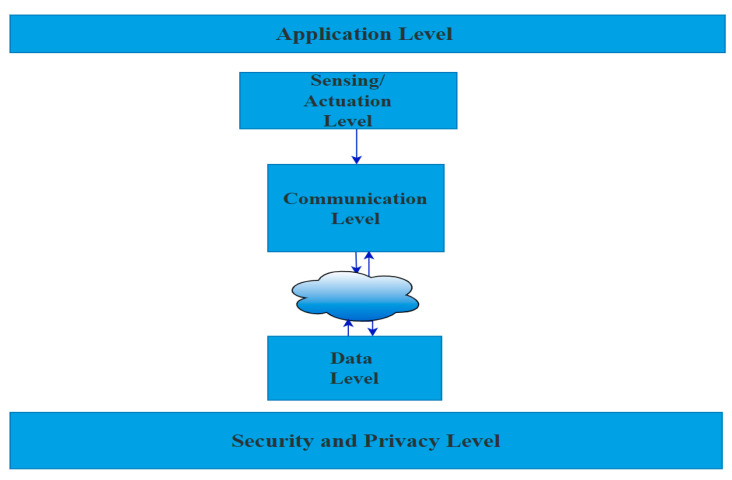
Block diagram plans architecture of smart grid applications.

The Internet of Things (IoT) has developed the conventional grid system into the modernized grid, called smart grid (SG). The traditional grid system establishes one-way communication from the grid to the house. However, SG implements bidirectional communication between the service provider and the distributed sensors. The Internet of Things (IoT) serves as a bridging component between sensing devices and the data plane in implementing the smart grid [1]. Figure 1 depicts the growing awareness of smart application cybersecurity, which necessitates implementing security and privacy assurance measures. The difficult work of meeting these demands has been delegated to the security and privacy level. As indicated in Figure 1, every individual level piece of this architecture requires a complete and effective security and privacy level. One of the main applications of SG with two-way communication is Advanced Metering Infrastructure (AMI) [2]. An important criterion in AMI application is to ensure security to serve several entities, such as domestic or non-domestic users and governments [3,4]. The absence of an effective security scheme makes communication between legal entities vulnerable to communication attacks [5,6]. One of the major constraints in providing security to IoT protocols used in sensor networks is the restricted resource availability of smart sensors.

The Constrained Application Protocol (CoAP) protocol is designed with resource-constrained sensors for the IoT application layer [7]. A Datagram TLS (DTLS) is recommended to make the CoAP secure [8]. The DTLS offers authentication, key exchange, and protection of communication between legal entities. However, DTLS is not a specific design for IoT applications, especially SG. Therefore, there are several problems while connecting it with the DTLS/CoAP over AMI directly. The DTLS/CoAP has to exchange six flight handshake messages between sensors. It fragments the packets into the 127-byte, but it leads to data loss and delays in communication [8,9]. The small packet size increases the number of packets, leading to network traffic due to frequent handshaking and packet loss. The large size eliminates the frequent handshaking process and unnecessary packet loss. Another issue associated with DTLS protocol is the chance of transmitting several Hello messages to a server. It leads to Denial-of-Service (DOS) attack against the server. The possibility of a DoS attack in DTLS/CoAP architecture increases bandwidth usage and allocation of resources for malicious Hello messages. Moreover, the periodic request message size in DTLS/CoAP is 32 bytes. The 32 bytes of the packet header and the energy reading information in Advanced Metering Infrastructure (AMI) tend to have large packet sizes. If congestion occurs during the communication, the gateway may receive the delayed packet or only a few packet fragments. In such a case, the gateway requests the smart sensors to retransmit the packets. It contributes to high communication delay, unnecessary packet retransmission, network collision, and energy consumption.

Thus, the proposed work plans to integrate the important features of DTLS in CoAP instead of using DTLS in a separate channel. The DTLS feature integrated with CoAP architecture is DF-CoAP. Moreover, the malicious nodes can misuse the DF-CoAP. Moreover, AMI sensors expect complete anonymity to their information. However, the AMI is a critical structure, and it does not desire to provide complete anonymity. For instance, law authorities must track users who attack the smart grid. Thus, conditional privacy preservation concealment of the identity information of a smart sensor is desirable in the sensor network. By considering the issues above in AMI, providing a lightweight security and authentication scheme becomes one of the research focuses in AMI.

A widely used cryptography technique to provide end-to-end security in CoAP is Advanced Encryption Standard (AES). The AES with 128-byte key size as a basic cipher suite of DTLS helps solve the issues of packet fragmentation, loss, and delay in the IoT environment. However, there is a limitation in applying the AES technique directly for secure data communication in DF-CoAP architecture for resource-constrained smart sensors. The AES generates different words using the original key, but the possibility of deriving the relationship between those words deduces the security level of AES. Furthermore, biasing inputs in the keyspace of AES creates the possibility of observing differences between the words in the ciphertext for malicious sensors. Thus, the proposed work plans to optimize the security using a lightweight and strong cryptography technique, AES, while minimizing its complexity. From the above discussion, this paper proposes a lightweight and secure authentication scheme for distributed sensor networks. The major contribution of this paper includes the following:

1. The proposed work aims to integrate optimised DTLS Features in default CoAP (DF-CoAP) and implement the lightweight authentication mechanism with DF-CoAP to ensure secure communication between authorised entities in a distributed sensor network.
2. The proposed approach uses the payload encryption-based handshaking process to reduce the handshaking process from three to two round trips. It ensures a lightweight and secure authentication scheme on the application layer.
3. By adding the NACK-based retransmission scheme to the DF-CoAP, the exact missing data in the flight is informed to the sender with the received byte to reduce the communication load.
4. The proposed work introduces the buffer size for intimating their exact size to the server and avoiding the buffer overflow in DTLS. It optimises the performance of DF-CoAP without affecting its security level.
5. The proposed work reduces the system complexity drastically by removing the duplicate features of the protocol version and sequence number component of the nonce explicit field from the packet header in DF-CoAP and introducing the parallel execution of S-boxes in SubBytes of AES.
6. The proposed scheme introduces the nonce and seed values to generate the secret and session keys dynamically, which significantly improves DTLS security.

### Paper Organization

The remaining part of this paper is organized as follows. Section 2 provides the related works on the lightweight authentication scheme for a distributed sensor. Section 3 provides the problem formulation, system (model) and attack model. Section 4 explains the proposed method with the figures and algorithm. Section 5 shows the performance results with detailed descriptions. Section 6 concludes the paper. 

## 2. Related Work

Device authentication is an imperative mechanism in verifying the entity identity of a node in a distributed sensor network and eliminates the impact of malicious behaviour. The Internet of Things (IoT) application layer protocol Constrained Application Protocol (CoAP) is used with Datagram Transport Layer Security (DTLS) for reliable and secure negotiation of a session, authentication, and data communication. Even though the DTLS provides a complete security model to CoAP, reducing energy consumption without compromising security is essential. The Advanced Metering Infrastructure (AMI) data are sensitive, and if the data are stolen, it results in significant economic loss to both users and power providers. Thus, the data transmitted over distributed sensors must be encrypted before being forwarded. The existing works mostly apply either symmetric or asymmetric encryption schemes. The former technique must share the secret key in advance, and the latter does not need to set a shared key in advance. However, the latter technique leads to more computational cost than the former encryption technique. Therefore, it is an effective method to generate a shared key and encrypt the data dynamically. Smart grid applications need to provide an effective authentication and security scheme in a lightweight pattern [10,11]. Since smart sensors are low-energy devices, highly complex security schemes that require more energy do not apply to smart sensors.

### 2.1. Authentication Schemes for Smart Grid Application

A security protocol is developed for the smart grid using different double auction mechanisms and homomorphic encryption [12]. This protocol provides authentication, security, and compatibility to smart grid technologies. It assigns pseudo-identity to each consumer in the smart grid and ensures the anonymity of sensors during communication. Moreover, the Paillier cryptosystem is used for encrypting the bids/asks. However, the homomorphic encryption schemes in [12,13] generate a lengthy cipher suite than the plain text, resulting in a large delay for encryption and decryption. A lightweight authentication and key agreement are suggested in [14] for a smart sensor network. For providing security, it exploits hybrid cryptography, i.e., Elliptic-curve cryptography (ECC) and a symmetric key. Before applying cryptography, both gateway and smart sensors are authenticated mutually. The hybrid cryptography-based security scheme protects against many attacks, but it lacks to focus on many privacy features, such as unforgeability and undetectability. Several ECC-based authentication schemes [15,16] have been proposed for the sensors network. However, they do not satisfy all the security features in sensor networks. The self-certified key distribution scheme eliminates the necessity of certificate management overhead [15]. However, using keys with small sizes does not always ensure security. 

A secure key agreement model for smart metering communications is developed in [17]. It eliminates the necessity of a secure channel during the registration of the entities and eliminates the Denial-of-Service (DoS) attack possibility. However, the size of the encrypted message is large and increases the system complexity. The Password-based Anonymous Lightweight Key agreement framework (PALK) is developed and improved in [18,19], respectively. They developed using ECC and symmetric hash functions. The superficial ECC operation performed in PALK with the multiplication of two points over the curve leads to incorrect authentication. Thus, the PALK is improved using block cipher-based encryption/decryption operations. An improved authentication protocol using ECC is developed in [18] for the smart grid environment. It utilizes the advantages of biometric information to improve the strength of ECC. However, small size keys for various security levels affect the performance of the authentication scheme. 

A pairing scheme between smart sensors and a server is expensive, and it does not apply to smart sensors with low power. In [20], a Certificate Less Two-Party Authenticated Key Agreement (CL2PAKA) scheme is suggested for smart grid applications. The CL2PAKA does not need to perform any pairing operation, and it implements only four scalar multiplication operations on ECC. The main disadvantage of certificate-less authentication schemes [20,21] is that the identity information cannot provide a public key for a long time. The identity of sensors alone is not sufficient to provide a public and secret key. In [22], a privacy-preserving architecture is suggested for the smart grid using a Q-learning-based optimised approach. It exploits the cryptography technique to outsource multiregional electricity data securely. It implements three dynamic protocols to perform primary operations in Q-learning, such as Q value updating, Q-learning training, and knowledge replaying with encrypted packet information. However, it consumes more time to reach an optimal value, which is less applicable to resource-constrained smart grid applications. 

### 2.2. Optimised Implementation of DTLS for CoAP

Several works have been suggested to improve the performance and security of DTLS in CoAP. In [23], an optimised implementation of DTLS for CoAP is suggested. The DTLS protocol is integrated with the CoAP and ECC optimizations which minimises ROM utilization. An optimization for DTLS is performed with connection-oriented communication and fragmentation. The CoAP message layer provides a connection-oriented communication, whereas the fragmentation is attained using the block-wise transfer feature provided by CoAP. Those mechanisms make the DTLS lightweight as well as strong in its security. The optimization technique applies a stateless cookie technique to reduce the resource consumption of smart sensors. This technique enforces the smart sensors to send Hello messages with the attached cookie again. According to the cookie validation, the server node can verify and decide whether to continue the handshake process. Even though the DTLS is used to secure and support the lightweight, CoAP lacks some security requirements. The DTLS fails in satisfying the important feature of CoAP, such as multicast communication. In addition, the DTLS handshake protocol can cause exhaustion attacks. It causes the nodes to lose their roles in the network and affects the entire IoT communication. Moreover, the security features used in DTLS cannot avoid replay attacks in all scenarios; thus, it is vulnerable to replay attacks and loss of resources among smart sensors. Moreover, the DTLS handshake protocol does not always ensure end-to-end authentication [24]. 

An optimised version of DTLS attempts to reduce the complexity and cost of communication by improving cookie exchange strength during the handshake process in CoAP [25]. During the connection establishment between the IoT sensor and server, several encryption concepts are integrated into the CoAP communication, and it provides an additional security layer to the CoAP. It introduces the cookie exchange technique for reducing the impact of the DoS attack. The sensor devices have to show their capabilities to the server before allocating the resources to the smart sensor. This assists in reducing the energy consumption of DoS attacks in IoT. The mechanism suggested in [26] reduces CoAP communication latency by applying the forward error correction. This approach assists in compromising the packet loss and communication delay. Consequently, it shows significant improvement in the throughput of IoT communication. Moreover, several attempts have been made to improve the efficiency and security of DTLS [27,28]. An enhanced DTLS in [27] separates the DTLS handshake and encryption phase to mitigate the delay, packet loss, and overhead of DTLS during the handshake process. Every smart sensor depends on the secure service manager to execute encryption and decryption of the data. 

The primary issue associated with the DTLS security is the possibility of sending several Hello messages to the server. A smart gateway-based authentication and authorisation method is suggested in [28] to provide security to health data communication. However, introducing the smart gateway is not always preferable in all IoT applications due to the induced overhead in the IoT environment. A flexible ECC-based CoAP attempts to establish a secure communication session between sensors and a server [29]. It solves the issues related to the key management and insecure communication channel. Even though the ECC algorithm exploits small-size cryptography keys, it increases the encrypted message’s size using such a key. Moreover, it is highly complex to implement in a resource-constrained IoT environment. The Diffie-Hellman (DH) key exchange and the certificate verification implemented in DTLS make it highly complex. A simplified handshake protocol of DTLS (DTLShps) is proposed to solve such an issue. It takes support from software-defined networking and mitigates the computational overhead of the smart sensors significantly. The controller generates symmetric and distributes keys to the communicating smart sensors. A powerful controller is used in the process of certificate verification. The controller replaces the DTLS server for exchanging the cookies with the DTLS sensor device. Furthermore, it exploits the BAN logic and Scyther tool to make the DTLS more secure. In [30], the grouping of handshaking messages is investigated instead of considering a single DTLS handshake message between sensors and a server. It drastically reduces the computational burden induced by the series of DTLS handshakes. However, there is a possibility for running Hello DoS attacks in the IoT environment.

A payload-based mutual authentication scheme is suggested to overcome this issue [31]. It avoids implementing DTLS through a separate control channel in IoT and reduces the possibility of attacks in resource-constrained sensors. Even though it introduces payload-based authentication in CoAP, data confidentiality is still achieved using AES. However, the traditional AES needs to share a secret key securely. Moreover, the utilization of secret keys for a long time reduces the security of CoAP messages in IoT. Thus, the traditional AES algorithm needs to be improved with dynamic key sharing and lightweight encryption processes.

### 2.3. Lightweight Cryptography Schemes for CoAP

Several lightweight encryption algorithms using symmetric and asymmetric techniques have been suggested for various IoT applications. A lightweight encryption algorithm is proposed in [32] to enhance secure data transmission of smart sensors. It exploits the Feistel structure and the network with a uniform substitution-permutation in a combinational form. Likewise, Data Encryption Standard (DES) in [33] applies symmetric key block cipher with Feistel structure. The plaintext of 64-bit and a key size of 56 bits are used in the encryption process with 16 rounds. However, the DES algorithm’s main drawback is its flexibility in Feistel structure and does not support any modification to support various IoT application services [34]. In [35], a symmetric encryption algorithm exploits multi-cloud computing and provides privacy in forwarding and backward directions. However, there is a possibility of information leakage. It provides fast response than DES, but it is vulnerable to various attacks. Moreover, the combination of several components in M-SSE makes it very complex and costly. Much valued logic and variable block length are involved in the encryption algorithm in [36], and it is performed in five rounds. Each round performs different operations. The first round performs the gamma and permutations procedures, and other rounds perform substitution and gamma procedures. Super-encryption cryptography in [37] combines the International Data Encryption Algorithm (IDEA) and Word Auto Key Encryption (WAKE) algorithm. The super encryption technique involves two or more symmetric cryptographic algorithms to ensure more security to IoT data. Even though they are designed specifically for resource-constrained smart sensors, they involve complex cryptography functions to provide high-level security. The minimum memory utilisation level and feasibility in implementation make the Tiny Encryption Algorithm (TEA) famous. The main TEA and its numerous developed versions apply the same key for encryption rounds. However, it degrades the strength and efficiency of the security algorithm. In addition, it takes a huge time for the encryption and decryption process [38,39]. A novel tiny symmetric encryption algorithm (NTSA) is suggested to improve the security of IoT networks by providing additional key confusion for each round of encryption [40]. However, the cryptography described above techniques is limited to providing the key length and security for IoT communication. However, the AES can handle an entire block of plain text in the form of a single matrix with the operation of substitution and permutation [41]. However, optimizing the AES algorithm to support resource-constrained smart sensors is still lacking in IoT.

## 3. Preliminaries

**Figure 2:**
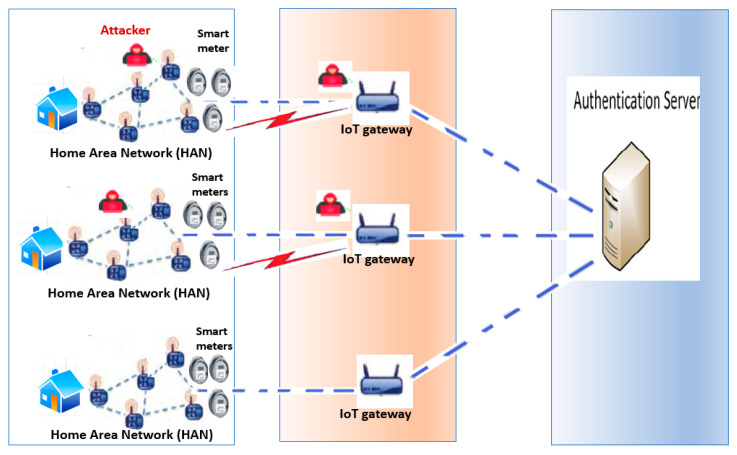
System model.

The Advanced Metering Infrastructure (AMI) network is responsible for providing data on the quality of power and quantity of resource utilization at the smart sensor side. The smart sensors in AMI are embedded with low computational and storage capabilities. Moreover, they communicate with each other and server using low data rates and lossy radio channels. The AMI network needs to redesign a proper Datagram Transport Layer Security (DTLS)/Constrained Application Protocol (CoAP) architecture to offer lightweight, low-latency, and highly reliable communication from both meters to gateway and gateway to meters. However, unreliable communication links in AMI are still suffering from adversarial activities. The deployed application layer protocol must cope with the DTLS security and overhead to satisfy the AMI low-latency and high-reliability requirements. Figure 2 depicts a general overview of smart grid advanced metering infrastructure (AMI). The figure depicts an AMI network that is implemented using a wireless sensor network (WSN). Wireless sensors are embedded into smart meters, and they work in industrial and scientific bands. A traffic concentrator receives traffic from power meters and acts as a communication gateway between a WSN network and server.

### 3.1. Problem Statement 

The design of DTLS is not suitable for resource-constrained smart sensors due to the expensive handshaking and lengthy cipher suite agreement process. Moreover, complex handshake processes are not always protected from attacks. Sending a handshake request message in DTLS to low-memory and low-capacity sensors looks like a Denial of Service (DoS) attack in IoT. An attacker could send several Hello messages to a server. This scenario would cause a DoS attack against the server. A loss or delay of the single fragment in DTLS enforces the whole flight to be retransmitted. In DTLS, the recording layer occurs 13 bytes of overhead. There are some duplicate parameters maintained in the recording layer, such as protocol version and sequence number. The DTLS allows the server to specify the max fragment size of smart sensors, but the server cannot decide the fragment size per the available sensors’ buffer size. Thus, the proposed work plans to solve the issues above and improve the DTLS security while minimizing its complexity.

### 3.2. Problem Formulation 

To construct the CoAP security model based on DTLS architecture, the sensor output (y) can be formulated.

(1) y=DTLS(s)−DTLS(o)

y represents the quality of CoAP/DTLS architecture in terms of both security (s) and overhead (o). Basic DTLS performs a handshake between communicating sensors for a long time. The notation Hd denotes the handshake duration.

(2) $$\mathrm{Hd}={Τ}_{\mathrm{session}}+{\;Τ}_{\mathrm{challenge}}+\Xi_{\mathrm{Processing}}$$

From the above equation, the Hd is estimated using the summation of round-trip time taken by session initiation request$,\;{\;Τ}_{\mathrm{session}}$, the round-trip time spent by smart sensor response and request,${\;Τ}_{\mathrm{challenge}}$, and the processing time of a request at the smart sensor, $\Xi_{\mathrm{Processing}}$. 

The processing time is increased with the number of redundant features appended in the DTLS messages. It may exceed the response timeout value and initiates the handshaking process again. Moreover, a delay of the single fragment in DTLS enforces the whole flight to be retransmitted. It is another factor behind the increase in DTLS (o).

(3) $$\Xi_{\mathrm{Processing}}\;\propto \;\mathrm{DTLS}\left(o\right)$$

The handshake phase explores either symmetric-key or asymmetric-key-based cipher suite to ensure the DTLS(s). The cipher suite selection makes a big difference in the value of y. If the symmetric key-based cipher suite is used, for example, Advanced Encryption Standard (AES), performance must be improved. However, sharing a secret key in advance and keeping it secret during communication against several attacks is a notable limitation. In addition, the delay induced by the AES algorithm makes smart sensors complex and increases energy consumption. Thus, the proposed work designs a lightweight CoAP authentication scheme by appending the important DTLS features and optimizing the AES algorithm successfully.

## 4. Materials and Methods

### 4.1. System Model and Network Model

The system model of the distributed sensor consists of the server, δ, which is situated in the corner of the IoT network, and N numbers of smart sensors. The server δ is involved in collecting and analysing data from N, so it is called a gateway. Based on the collected data, δ provides predictive information to sensors. The N smart sensors are located in different positions in the network to mimic the sensors. Two-way wireless communications led to several potential vulnerabilities. For instance, 

(i) There is a possibility for compromising the smart sensors connected to the server by unauthorized users. 

(ii) By injecting false data or implementing Denial of Service (DoS) into Advanced Metering Infrastructure (AMI), the malicious users can trip the energy supply to various locations from the smart grid. 

(iii) Smart home energy sensors need to send consumption usage periodically to δ, but the malicious users may leak consumption data by eavesdropping on the communication channel, and it affects the consumer’s privacy. 

To avoid such attacks, the proposed lightweight Constrained Application Protocol (CoAP) authentication scheme involves the following processes.

**Definition** **1** **(Session** **Initiation).** *During the registration phase, a gateway δ provides a unique and fixed bit-length identity ID along with the pre-shared secret key (ψ) and seed value, Se to N sensors separately. Both server δ and N sensors require to generate the ψ and session key,* *ϒ* *dynamically for each communication process using the pre-shared secret values. Thus, the gateway* *δ* *shares* *ψ* *i and Se with the corresponding sensor Ni *via* a secure channel. Even though it is essential to provide an efficient security scheme for sensor network communication, law authority needs to track who perform malicious activities in the smart sensor. Thus, conditional privacy preservation, the concealment of the identity information of a smart sensor is desirable. Thus, the randomly chosen pseudonym ID (P-IDi) replaces the original ID of sensors. The secret values ψi, Se, and P-IDi are known to the consumer Ni and the δ. Hence, other users and attackers cannot obtain the real IDi. Therefore, the proposed lightweight security scheme provides identity privacy without compromising communication security.*

**Definition** **2** **(Server** **Challenge).** *After receiving the CoAP request message from Ni, the δ compares the ψi value of the corresponding node Ni. If the secret values are matched, the gateway δ believes that Ni is an authorized sensor device, and it sends the server challenge message with the nonce value 1, and both the entities such as Ni and δ derive the secret values dynamically.*

**Definition** **3** **(Smart** **Sensor** **Response** **and** **Challenge).** *The proposed lightweight authentication scheme authenticates the sensors on both sides using two nonce values, ψ, andϒ*.

Dynamic ψ, Dψ = ψ || Nonce 1 || Sei for mutual authentication.

At this stage, the smart sensor generates an encrypted payload. An Exclusive OR (XOR) operation is performed on the message using Dψ. Here, the message consists of Nonce 2.

**Definition** **4** **(Server** **Response).** *The gateway node δ needs to decipher the encrypted payload to retrieve the Nonce 2 value and generate the session key dynamically.*

Dynamic ϒ, Dϒ = ϒ || Nonce 2 || Sei for secure communication.

**Definition** **5** **(Secure** **and** **Optimised** **Communication** **in** **IoT).** *The Datagram Transport Layer Security (DTLS) Feature integrated with CoAP (DF-CoAP) exploits six flight messages, leading to communication delay and energy inefficiency. Thus, the proposed work applies the payload-based mutual authentication scheme and successfully reduces the number of flight messages from six to four. During secure communication, the packet header removes the protocol version and the sequence number component of the nonce explicit field. Moreover, the ACK message includes the exact missing data in the current flight together with the right-most received byte.*

### 4.2. Overview of the Proposed Scheme

**Figure 3:**
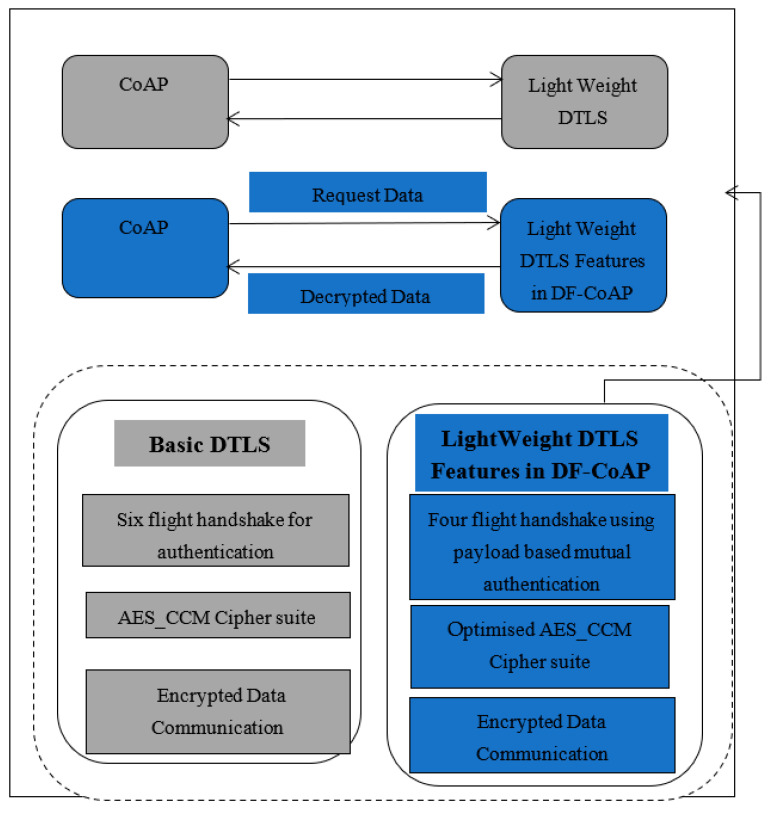
Block diagram of the proposed work.

The proposed lightweight PEOS authentication scheme in DF-CoAP verifies the identities of sensors and the server in two-way communication. A proposed lightweight handshake mechanism including only two round-trip message exchanges is used for two-way authentication in the sensor network. As shown in Figure 3, both the challenge in sensors and the server for two-way authentication generate encrypted payloads with some optimised DTLS features. It assists in improving the performance and overhead of a payload-based authentication scheme. Moreover, the encryption scheme Advanced Encryption Standard-Counter with Cipher block chaining Message authentication code (AES-CCM) is optimised by deciding the methods from design choices for AddRoundkey, SubBytes, ShiftRows, and MixColumns and dynamically selecting the secret key using a nonce, seed, and secret master key.

Lightweight DF-CoAP for Sensor network: DF-CoAP and AES-CCM provide flexibility in cipher suite usage and design choices. Using the features of DF-CoAP, the payload-based four-way handshaking and missing data retransmission using NACK make the DF-CoAP lightweight for sensor networks. 

Payload Encryption-Based Four-Way Handshaking with Unique Packet Features: It explores the encrypted payload-based mutual authentication scheme, but the limited payload space in sensors makes it unsuitable for larger payloads. Existing works attempt to reduce the overhead of CoAP using four-way authentication schemes. However, the nature of DTLS flight messages, the exact missing data in the current flight, is unknown to the sender and a loss or delay of a single fragment in DTLS enforces the whole flight to be retransmitted. Thus, the proposed scheme aims at optimizing the payload-based mutual authentication process using exact buffer size intimation and missing data retransmission schemes. 

Optimised and Secure AES-CCM Cipher Suite for Distributed Sensors: Applying the existing AES-CCM for secure data communication in sensor networks is inadequate for resource-constrained sensors. There are design choices for the processes used in AES-CCM. As per the features of smart sensors, selecting appropriate methods for such a cryptographic scheme improves the performance of a proposed lightweight DF-CoAP scheme. 

Moreover, parallel execution of SubBytes and multiple small s-boxes effectively reduce the time delay and system complexity. The dynamically generated secret key in two-way authentication is used as the symmetric key for optimised AES-CCM. Thus, the proposed mutual authentication scheme reduces the round-trip time of CoAP smart sensor challenge messages without compromising the communication security in distributed sensors. 

### 4.3. Lightweight DF-CoAP for Distributed Sensor Network Using Payload Encryption-Based Four-Way Handshaking 

The proposed lightweight PEOS scheme in DF-CoAP architecture provides authentication using the payload of messages exchanged among the sensors and server. Both the consumer and the server challenge each other during the authentication process. The two-way authentication scheme uses four handshake messages with the improved DTLS features messages with exact buffer size and missing data re-transmission scheme using the NACK method. 

**Figure 4:**
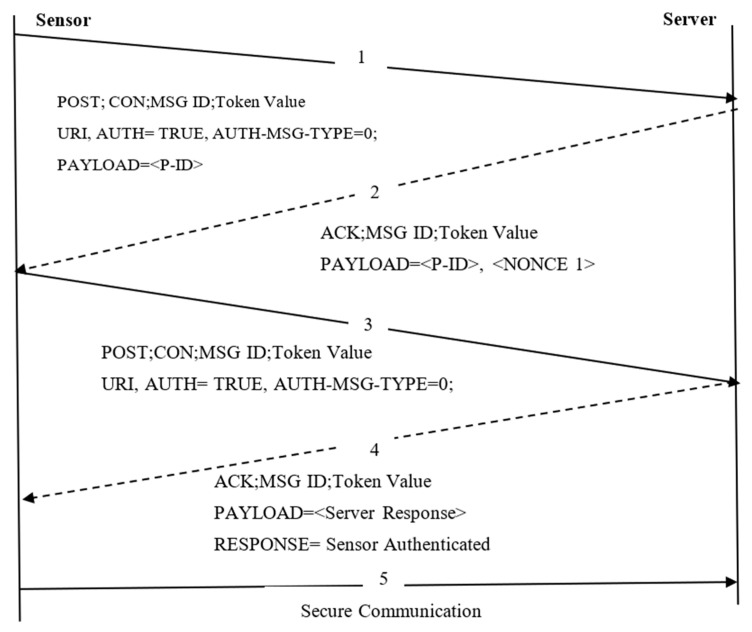
Two-way authentication scheme using payload encryption.

As shown in Figure 4, four steps are involved in the proposed work.

1. Session Initiation
2. Server Challenge
3. Smart Sensor Response and Challenge
4. Server Response and Secure Communication

**Session Initiation and Server Challenge:** The session initiation is a provisioning phase. It is a prerequisite offline phase, where the sensor, N share a 128-bit AES pre-shared secret key (ψ) and seed value, S_e_, with the server, δ. The pre-shared secret values are known only to the corresponding sensors and server. Each sensor has a unique identifier (P-ID), and the P-ID_i_ is used for identity verification on the server-side. Upon successful verification, both parties communicate with each other to exchange the session key. It is assumed that the sensors used on the sensor side are temper-safe, and they can alert the data by generating an alarm if an attacker tries to tamper with the sensor. Each sensor sends a request message to the server. To create a session, this request message acts as a confirmable message (CON). A challenge message is created at the server with a specific token for correlating the request with a corresponding challenge when the secret values are matched. The server challenge message consists of Nonce value 1, and it assists that both the entities such as N_i_ and δ derive the secret values dynamically.

(4) PAYLOAD(〈P−ID〉, 〈NONCE 1〉)=OP_AES−CCM { Dψ, (Ψresultant)}

where

(5) $$\Psi \mathrm{resultant}\;=\mathrm{XOR}\left(\psi ,\mathrm{NONCE}\;1,{\;S}_{e}\right)$$

Exact Buffer Size in Hello Message Transmission: The server specifies maximum fragment lengths for each smart sensor and allows data transmission within a specified fragment length. The undefined buffer size incurs buffer overflow and data loss. If a buffer with a fixed-length overflows in a node, the data stored in the memory is overwritten. An attacker can use it to send frequent requests to the nodes and destroy the network. Moreover, the DTLS max fragment length mechanism is not symmetrical since the server cannot state the buffer size of nodes exactly. Instead of a server, allowing resource-constrained sensors to advertise their maximum fragment length lowers the possibility of data loss and buffer overflow issues. The proposed work allows the nodes to append the field of Exact Buffer Size (EBS) in Hello Message to avoid such a problem. 

Smart Sensor Response and Challenge: The Nonce 2 value the message payload during smart sensor response and challenge. Two options, i.e., Auth and Auth-Msg-Type, denote the type of operation executed on a resource at δ. During the second phase, the server δ obtains and verifies the object ID from the message payload. If it verifies the identity, the δ responds with an encrypted payload using the optimised AES-CCM algorithm. It supports creating a new session with a response code of 2.01 (Created). This phase generates the dynamic secret key, Dψ using the nonce value 1, ψ, and Se_i_. Using such Dψ, the payload with Nonce 2 value is created at the smart sensor-side.

(6) PAYLOAD(〈P−ID〉, 〈NONCE 1〉)=OP_AES−CCM { Dϒ { Dϒ, (Ψresultant 1)}}

where

(7) $$\Psi \mathrm{resultant}\;1=\mathrm{XOR}\left(ϒ,\mathrm{NONCE}\;2,{\;S}_{e}\right)$$

Even though the overhead of DTLS is not suitable for resource-constrained sensors since the DTLS record layer header appends 13 bytes of overhead, some of the fields carried in the header are unavoidable, and other parameters are redundant. It is because they are included for backward compatibility reasons. It becomes substantial for the resource-constrained networks. The DTLS feature integrated with CoAP architecture (DF-CoAP) header fields that are not strictly needed can be removed to reduce the burden. The proposed work removes the fields of the protocol version and the sequence number component of the nonce explicit field from the layer of OP_AES-CCM. The second field is a duplicate of the sequence number used in the DF-CoAP. It assists the proposed work to eliminate 8-bytes per record. An algorithm for CoAP message exchange using the proposed work is given in Algorithm 1.
**Algorithm 1.** Secure Authentication and Communication.**1: Session Initiation:** (a) Each Sensor N_i_ is provided with a unique P-ID_i_, ψ_i_, and seed value, S_e_ (b) A gateway δ is provided with all P-ID_i_, ψ_i_, and seed value, S_e_ stored in an array A[ ][ ][ ] 2: **Step 1 [Session Initiation]:** [Input: (P-ID_i_, ψ_i_, and seed value, S_e_)] 3: *for* i = 1: N do (Nested For loop generates a three-column server table) 4: for j = 1: 3 do 5: input (A[i][j][k]) (P-ID_i_, ψ_i_, and seed value, S_e_ of N_i_ are stored in the array) 6: *end for* 7: *end for* 8: N_i_ sends a CON message containing P-ID_i_, ψ_i_, and S_e_ in the payload to δ 9: **Step 2 [Server Challenge]:** δ retrieves P-ID_i_, ψ_i_, and S_e_ to authenticate the received CON message 10: *if P-* ID_i_, ψ_i_, and S_e_ == A[i][j][k] then a node i is authenticated successfully 11: Session Created 2.01 12: **Step 3 [Sensor Response and Challenge]:** δ responds with an encrypted payload, with P-ID and NONCE 1 value.13: *else* 14: N_i_ Unauthorized 4.01 15: *end if* 16: Step 4 **[Sensor Response & Challenge]:** N_i_ deciphers challenge and responds with anencrypted payload, OP_AES-CCM {NONCE 2} after verifying the δ using Dψ 17**: Step 5:** Ni compares generated Dψ using NONCE 1 and retrieved.Dψ from N_i_ 18: *if* Both matches then 19: S is authenticated 20: **Step 6 [Server Response]:** δ generates Dϒ and verifies the sensor challenge 21: *if* Both matches then 22: [Access Granted]- Ni Authenticated 23: **Step 7:** δ responds as Sensor Authenticated 24: *else* 25: [Access Denied]-Ni Unauthenticated 26: *end if* 27: **Step 8 [Data Exchange]:** Mutual data exchange between δ and N_i_ take place. 28: *else* 29: δ is unauthenticated 30: *end if*

Server Response and Secure Communication: Finally, the server deciphers the encrypted payload using the same session key generated at the smart sensor-side in the server response phase. The derived session key is used to implement secure communication between sensors entities successfully. The communication between the server and smart sensors should consider the EBS field to reduce buffer overrun and data loss.

**Figure 5:**
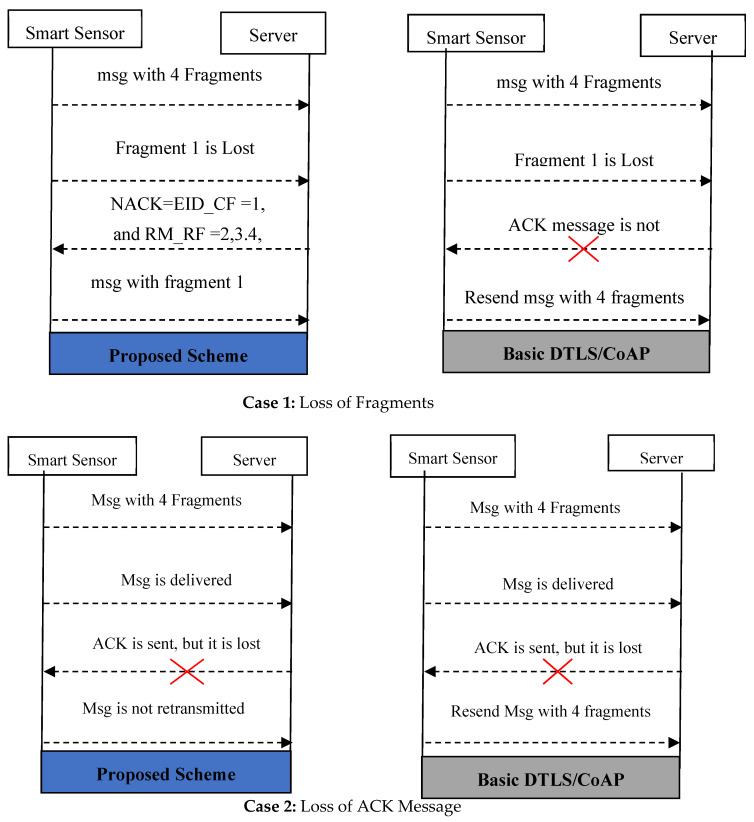
Communication between server and smart sensors.

Missing Data Retransmission using NACK without Encouraging DoS Attack: CoAP data transactions uses the observe model in Figure 5. The CoAP is modelled by improving the 6LoWPAN fragmentation in IoT [42]. The fragments are the traditional fragments that result from fragmenting network layer packets. The fragmentation breaks packets into smaller pieces in an adaptation layer that allows IPv6 datagrams to meet the requirements of the IEEE 802.15.4. As per the proposed work, the data fragmentation is also adopted and improved by the application layer protocol. A single fragment loss or delay forces the DF-CoAP to retransmit the whole flight in the resource-constrained sensors, as per the DTLS. The proposed lightweight authentication utilizes the Non-Acknowledgement (NACK) packet to avoid retransmission of the entire flight in case of losing a single fragment. An Acknowledgment (ACK) is used to confirm the receipt of all flights in DF-CoAP. In case of indicating that a packet has been lost, corrupted, or delayed and to resend it, the DF-CoAP sends NACK report to the transmitter nodes. The DTLS handshake associates every fragment with a unique identifier. The proposed work utilizes such identification in the NACK report and obtains the exact identity of the missing data in the current flight along with the right-most received byte. The nodes retransmit only the lost or delayed fragment with this identity information instead of sending the whole flight. There are two fields in the NACK report: the Exact IDentity of the missing data in the Current Flight (EID_CF) and the Right-Most Received Flights (RM_RF). If the EID_CF is assigned as null, the retransmission does not occur on the consumer side. In such a case, the RM_RF denotes the identity of entire flights. Otherwise, only a mentioned identity of flight in EID_CF is retransmitted from the consumer node. Utilizing the NACK, the proposed lightweight authentication scheme avoids unnecessary retransmission of entire flight messages to lose a single fragment. Figure 5 compares the retransmissions of default DTLS and the proposed scheme. Case 1 considers that a server cannot obtain the entire flight message due to the loss of the first fragment. After sending the NACK with EID_CF = 1, and RM_RF = 2, 3.4, the consumer node resends only fragment 1 in-flight message. However, the basic DF-CoAP resends an entire message as it does not receive the ACK message from the server. The second case differs from the first one. The ACK message is lost due to any network conditions. In such a case, the smart sensor resends the packet as per basic DTLS, but the proposed scheme does not resend the packet without receiving the NACK message.

## 5. Attack and Cost Analysis

The proposed Payload Encryption-based Optimisation Scheme for lightweight authentication (PEOS) authentication scheme ensures secure communication while implementing on Constrained Application Protocol (CoAP) over the Internet of Things (IoT). It can handle various attacks and secure communication with mutual authentication between the server and smart sensors. The possibility of security provision against different attackers in IoT applications is discussed below.

Security against Replay Attack: Security against Replay Attack: An attacker generally fails to change the messages between the smart sensors and server in a strong cryptographic mechanism. However, an attacker can copy a valid message with a wireless communication protocol and resend it to the server. If an attacker node A_i_ traces a session initiation request for a legitimate IoT sensor device, it can later gain network access. The replay attack is failed to attack the proposed PEOS because all entities generate Nonce values to prevent the attacker from sending the packets again. The nonce values are generated randomly, and it is varied over communication. If a node replays the old packet, the server and sensors identify the attack packet using the expired nonce values.

Security against Guessing Attack**:** Another main type of network attack is the secret key Guessing attack. The legitimate smart sensors, which have access rights to a server, are compromised by identifying the identity and secret key of the legitimate smart sensor. The secret key guessing attacks can be classified into brute force attacks and dictionary attacks. The brute force attacks attempt to analyse every possible code, overheard message, and secret key until it finds the correct one. This type of attack takes a long time to complete. A dictionary attack is another type of secret key guessing attack. It explores a dictionary of common secret keys to identify the exact secret key of legitimate smart sensors. If attacker A_i_ penetrates the server challenge between S_i_ and δ over a long time, it attempts to guess the ψ. If it is traced, an attacker can access the network as a legitimate sensor device. An attacker fails to identify the secret key ψ of any IoT sensor device because it knows the randomly chosen nonce value and seed value. Moreover, dynamically generated passwords using random generators periodically cannot be identified by brute force and dictionary attacks. As a result, it ensures security against guessing attacks on smart sensors in IoT applications. 

Security against DoS Attack**:** Denial of service is accomplished by flooding the target smart sensor with superfluous CoAP requests and overloading the server to prevent some or all legitimate requests from being transmitted to the server successfully. In a distributed DoS attack, the incoming traffic flooding is originated from different sources. 

An attacker has to overhear the CON message to implement the DoS attack against PEOS because the DoS attacks send the same request multiple times for destroying the target sensor. According to the PEOS, the smart sensor uses a token to identify the fresh request messages and rejects login requests containing the same token value and Nonce values again.

Security against Traceability Attack**:** A traceability attacker traces several requests to identify the IoT sensor device identity and secret data. However, the PEOS explores the pseudonym identity instead of the original one and ensures security against traceability attacks. Moreover, the proposed scheme ensures better data confidentiality, even in the worst case of initial key compromise, because it uses AES with a dynamic key-generation scheme. 

## 6. Experimental Evaluation

**Figure 6:**
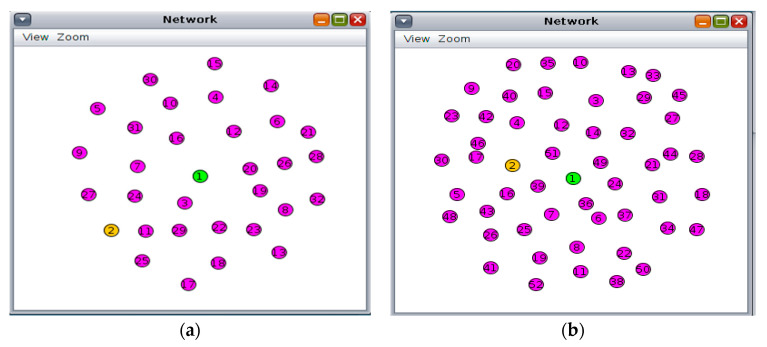
Experiment scenarios based on Contiki OS. (**a**) Network design diagram for 30 nodes; (**b**) Network design diagram for 50 nodes.

The proposed Payload Encryption-based Optimisation Scheme for lightweight authentication (PEOS) is implemented on the DTLS feature integrated with CoAP architecture (DF-CoAP) to demonstrate its performance using the Contiki Cooja network simulator. The communication process is conducted between the smart sensors and the gateway. However, adversarial activities disturb the sensor’s data communication. The lightweight mutual authentication scheme, PEOS, is applied to the DF-CoAP to ensure secure communication between smart sensors and the gateway. This section demonstrates the experimental results of the PEOS, existing Payload-based Mutual Authentication (PbMA) [30,31], and DTLS/CoAP [7,8]. As shown in Figure 6, the experiment is conducted on 30 and 50 compatible smart sensors, one border router, and one server working under the control of the Contiki OS for 300 s. In the simulation, two types of motes are used for simulation: for border–router, the z1 mote type is used, and for sensor and server nodes, the wismote mote type is used. The simulation is conducted on various numbers of smart sensors to identify the impact of network scalability on the efficiency of lightweight authentication schemes. The simulation is performed in a 100 × 100 m^2^ area for both proposed and existing works. In the distributed sensor network, the payload size is varied from 64 to 1024 bytes. Various scenarios are created with payload since the payload plays an important role in the efficiency of lightweight authentication. The communication range of each node is set to 50 m. Moreover, Table 1 describes the experimental parameters. 

### 6.1. Time, Computation, and Storage Complexity 

In addition to providing a security scheme, the lightweight authentication scheme used in PEOS is evaluated in terms of time, storage, and computational complexity. 

#### 6.1.1. Time Complexity

The time complexity of PEOS is estimated in the metric of handshake duration and communication. The handshake duration can be defined as the sum of time taken by two round-trip messages, including session initiation requests and the smart sensor response and challenge between smart sensors and a server. The smart sensor acknowledges the session initiation request through a smart sensor response, whereas the server acknowledges the smart sensor response and challenge through a server response. The time complexity, Time_Comp_ is computed at the smart sensor-end using the following equation.

(8) $${\mathrm{Time}}_{\mathrm{Comp}}=\mathrm{Hd}+T_{\mathrm{Communication}}$$

From the above equation, the Hd is estimated using the summation of round-trip time taken by session initiation request, the round-trip time taken by smart sensor response and request, and the processing time of a request at the smart sensor. Moreover, the summation of Hd and the time taken to deliver data denotes the time complexity of the proposed work. Instead of sending the messages through a control channel, both the existing and proposed schemes implement payload-based mutual authentication. Thus, both of them reduce the time complexity significantly [30]. However, the proposed work shows a noticeable reduction in time complexity compared to the existing work. Since the proposed work applies the NACK-based message transmission, it assists the PEOS to inform the smart sensor about exact missing data in the current flight with the right-most received byte. It reduces the number of retransmissions as well as Time_Comp_ in PEOS. 

#### 6.1.2. Computational and Storage Complexity

The computational and storage complexity is decided based on the number of encryption/decryption operations, signature/verification operations, and the random number generation. The proposed scheme shows a significant reduction in the computational cost of PEOS by secret key generation at the server and smart sensor-side individually. However, the existing payload-based encryption scheme needs to periodically share the secret key with each smart sensor [30]. Thus, the proposed work shows a significant reduction in computational complexity more than the existing work. A notation of α in the following equation shows a reduction in the computational complexity of the proposed work, compared to the payload-based encryption scheme, since it explores the based AES scheme. The parallel execution of S-boxes in SubBytes of AES reduces the complexity of the proposed work drastically. Moreover, the proposed scheme introduces the nonce and seed, but it does not significantly impact the complexity than the generation of secret key for each smart sensor at the server where α denotes the interval of refreshing the secret key for each sensor device at the server.

(9) $$\mathrm{Comp}\;{\mathrm{Complexity}}_{\mathrm{Existing}}=O(\alpha N)$$

(10) $$\mathrm{Comp}\;{\mathrm{Complexity}}_{\mathrm{PEOS}}=O(\alpha \mathrm{NlogN})$$

The storage cost is decided based on the number of parameters used for mutual authentication and secure communication process. The storage cost of the proposed scheme on the smart sensor side is smaller than the existing work due to the incorporation of dynamic secret and session key generation. On the server-side, both works show similar storage complexity. Thus, the complexity of PEOS is significantly reduced. Table 2 illustrates that the smart sensor-side storage cost is 384 bits, while the storage cost of the server is 640 bits.

#### 6.1.3. Performance Metrics

The simulation is conducted for evaluating the following metrics for lightweight payload-based mutual authentication, such as PEOS on DF-CoAP, Payload-based Mutual Authentication (PbMA) [31], and CoAP/DTLS [8], by varying the number of smart sensors and payload size. 

Throughput: Number of bits transmitted successfully to a server per second.

(11) Throughput (bitss)=Total Number of Transmitted Packets∗8Simulation Time

In Equation (16), the packets are given in bytes, and so it is converted into bits by multiplying with 8. When the total number of bits is divided by the simulation time, the equation gives the number of bits transmitted to a server per second. 

Communication Duration: The communication duration is the time a smart sensor node takes to deliver a data packet successfully. Moreover, it includes the handshake process, mutual authentication, and secure communication with the server.

(12) Communication Duration (milliseconds)                             =(Total Number of Transmitted PacketsSimulation Time∗Node Count)  ∗1000 

Energy Consumption: The amount of joules consumed by a smart sensor to deliver the data packets to the gateway.

(13) Energy Consumption (Micro Joules)                              =(Total amount of consumed energy in the network over simulation timeNode Count )                              ∗ 1,000,000  

Message Size Overhead: It is defined as the total length of the header in the packets transmitted and measured in bytes.

(14) Message Size Overhead (Bytes)=Total length of the Packet−Length of Payload

### 6.2. Experiment Results for 30 Node Topology

**Figure 7:**
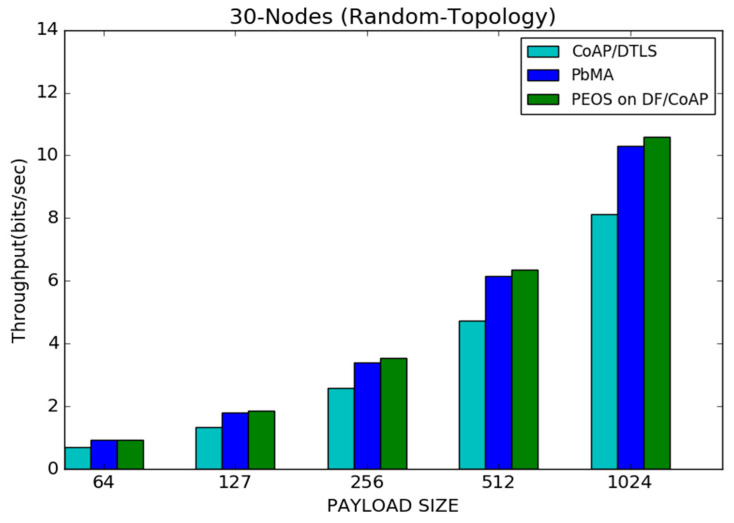
Payload size vs. throughput for 30 node topology.

In the IoT topology, 30 nodes are positioned to simulate the CoAP communication and to compare the performance of the proposed and existing schemes. With reduced payload size, more packets can be transmitted in the network. High network traffic interrupts the device process and tends towards high packet loss. It is the reason behind the throughput improvement in all the works with the payload size, shown in Figure 7. For instance, the proposed scheme attains nearly 2 bits/s of throughput with 127 bytes, and it is increased to 6.4 bits/s and 11 bits/s with 512 and 1024 bytes of payload, respectively. Likewise, CoAP/DTLS improves the throughput from 0.8 to 8 bits/s, and the payload-based existing scheme improves the throughput from 1.8 to 6 bits/s, when the payload is increased from 64 to 1024 bytes. The proposed scheme outperforms the payload-based mutual authentication and base DTLS under various payload sizes, as shown in Figure 7. The proposed authentication scheme decides the exact buffer size for each smart sensor and reduces unnecessary packet loss. However, the basic DTLS scheme [8] allows the server to randomly decide the buffer size, reducing the throughput compared to other schemes. When increasing the payload size from 127 bytes to 1024 bytes, the difference of network throughput between proposed and basic DTLS starts to improve from 0.1 to 0.5 bits/s. Figure 7 shows that the proposed scheme performs better than all other existing schemes. 

**Figure 8:**
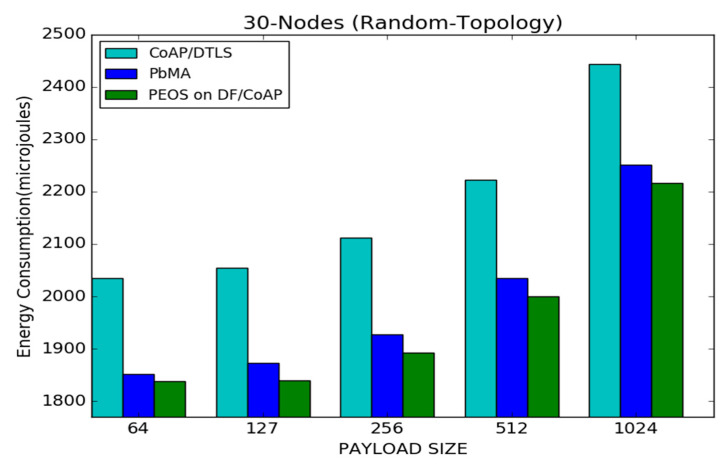
Payload size vs. average energy consumption for 30 node topology.

Figure 8 shows an energy consumption performance for the proposed and existing schemes on CoAP. The PEOS scheme outperforms the PbMA [31] and the basic DTLS scheme [8] when the network sends the data packets in various payload sizes. As with the payload size of 64 and 127 bytes, the proposed scheme achieves a reduction in the network traffic and no huge change in energy consumption compared to the basic CoAP/DTLS. The energy consumption in the proposed scheme increases slightly with the payload size. For instance, the proposed scheme increases the energy consumption from 1830 to 2200 micro joules with a payload size of 64 to 1024 bytes. The proposed and existing payload-based schemes spend less energy than basic CoAP/DTLS. Moreover, the difference in average energy consumption between the proposed and existing payload-based encryption schemes is minimal. For instance, the difference between the PEOS and existing PbMA is 25 micro joules with a payload size of 512 bytes. The proposed PEOS explores unique features of basic DTLS, resulting in a small amount of energy consumption. The concepts of exact fragment sending and delayed Mixcolumns reduce the requirement of additional storage registers and slightly reduce the energy consumption level than the existing payload scheme. However, the difference between the proposed and basic CoAP/DTLS is 230 micro joules with a payload size of 512 bytes.

**Figure 9:**
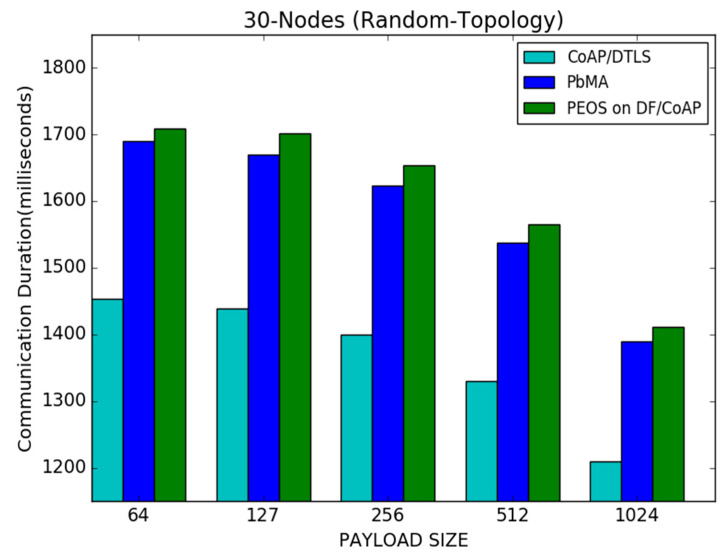
Payload size vs. communication duration for 30 node topology.

Figure 9 shows the results of communication duration for both the proposed and existing schemes. The proposed and existing payload-based encryption schemes, such as PEOS and PbMA, take more or less equal duration for delivering the data packets. For instance, with a payload size of 512 bytes or under less network traffic, the proposed and existing payload-based encryption schemes successfully transmit the data packets in 1200 milliseconds and 1500 milliseconds, respectively. However, the basic CoAP/DTLS delivers the packets in 1350 milliseconds. High packet loss in basic DTLS/CoAP is the main reason behind the small communication duration. As observed, since the proposed scheme delivers many transmitted packets due to the network traffic reduction using exact buffer size declaration and fragment transmission, the delay difference in the proposed scheme is considered to ensure low packets loss and retransmission. However, the existing schemes attain fewer throughput, and at the same time, take more time to deliver the data packets. For instance, in a payload size of 64 bytes, the proposed scheme takes a time of 1710 milliseconds. However, the PbMA and CoAP/DTLS take 1680 milliseconds and 1480 milliseconds, respectively. 

Generally, the CON CoAP messages increase the network delay. However, the proposed work can maintain the delay. The CoAP retransmission scheme in the proposed method is optimized using NACK-based retransmission. The payload encryption-based handshaking process reduces the handshaking process from three to two round trips. Thus, the delay is reduced considerably in the proposed scheme. 

**Figure 10:**
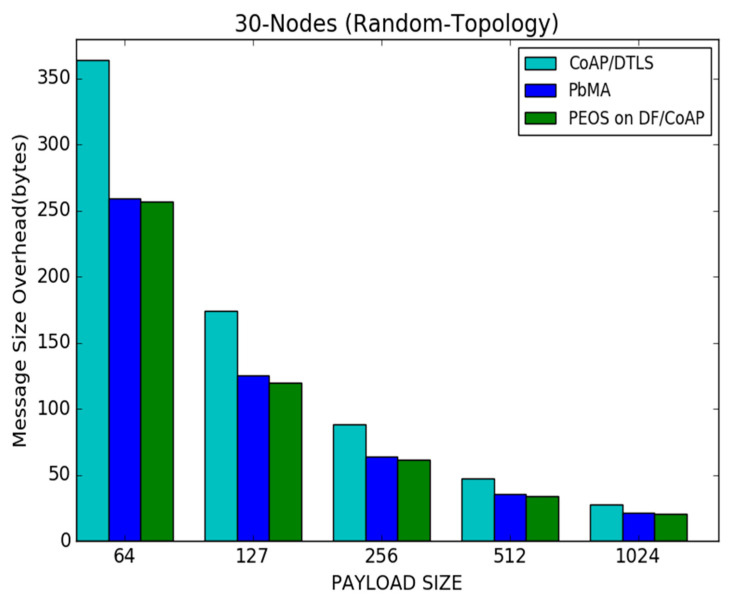
Payload size vs. message size overhead for 30 node topology.

Figure 10 illustrates the performance of the proposed PEOS and existing security schemes, PbMA, and basic CoAP/DTLS when the payload size is varied from 64 to 1024 bytes. Both the payload-based encryption schemes, such as PEOS and PbMA, reduce the message size overhead due to the utilization of unique DTLS packets. The proposed PEOS considerably reduces the packet header’s size and an unnecessary delay in communication. It improves the availability of network resources and the possibility of successful packet transmissions to the server. It reduces the message size drastically compared to other protocols. For a payload size of 127 bytes, the proposed PEOS scheme experiences a message size overhead of 120 bytes, whereas the existing payload-based authentication scheme, PbMA, and basic CoAP/DTLS experience a message size overhead of 130 bytes and 170 bytes, respectively. With an increment of payload size, all the authentication schemes reduce the number of messages. So the message size overhead, the total length of the header in all the packets transmitted is also reduced. In Figure 10, the proposed scheme experiences 120 bytes of message size overhead, whereas the message size overhead of the proposed scheme is reduced to 30 bytes with a payload size of 512 bytes. For a payload size of 127 bytes, the basic CoAP/DTLS scheme experiences 170 bytes, whereas it is reduced to 40 bytes when the payload size is 512 bytes. 

### 6.3. Simulation Results for 50 Node Topology

**Figure 11:**
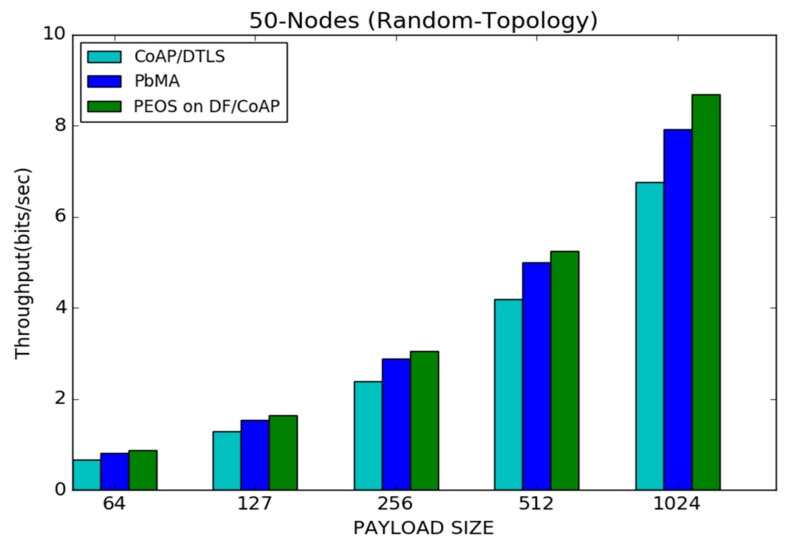
Payload size vs. throughput for 50 node topology.

Figure 11 represents the throughput for various schemes under 50 node topology. The network throughput is highly related to network traffic and congestion. The minimum value of payload size increases the network traffic and network congestion unnecessarily, leading to packet loss at routers. It is the main reason behind the throughput improvement with the payload size increment. The proposed and existing payload-based schemes append only the important features of DTLS and reduce the packet length, which improves the network throughput and reduces packet loss. For instance, the proposed scheme PEOS attains nearly 0.9 bits/s of throughput with 64 bytes, and it is increased to 8.7 bits/s with 1024 bytes of payload. Likewise, CoAP/DTLS improves the throughput from 1.2 to 4.1 bits/s, and the payload-based existing scheme improves the throughput from 0.5 to 6.3 bits/s when the payload is increased from 64 to 1024 bytes. Moreover, the proposed scheme improves the network throughput tremendously compared to the existing PbMA scheme and CoAP/DTLS. The existing schemes implement the whole flight retransmission scheme. It means that the single fragment loss in those schemes forces to send the whole flight again. Moreover, both the existing schemes explore pre-shared keys for a long time, increasing attacker activities and traffic. For instance, the proposed scheme works better than the existing payload-based scheme, PbMA, by 8.7% when the payload size is 1024 bytes.

**Figure 12:**
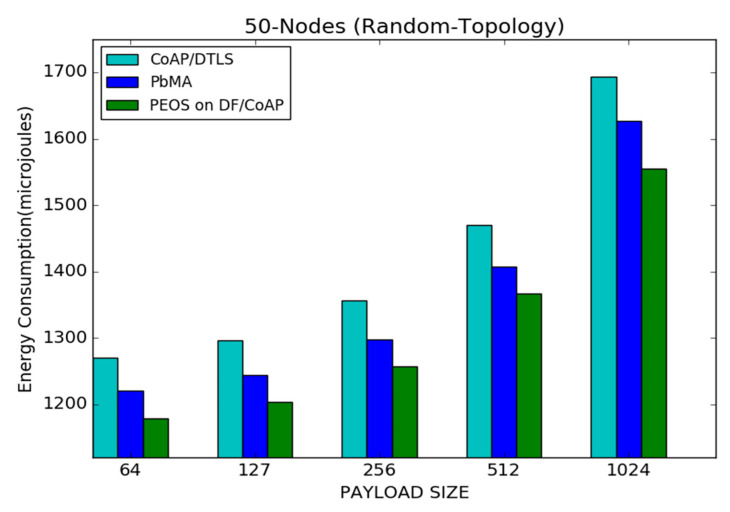
Payload size vs. average energy consumption for 50 node topology.

Figure 12 shows the influence of the payload size on the performance of the proposed and existing security schemes with CoAP, PEOS, PbMA, and basic CoAP/DTLS under 50 node topology. The proposed scheme demonstrates its performance with minimum energy consumption, especially with a small payload size. The proposed scheme reduces the number of transmitted packets with a large payload size, but the packet length is high. It increases the energy consumption slightly with the increment in payload size. 

For instance, the proposed scheme PEOS spends 1170 micro joules with a payload size of 64 bytes, whereas it is increased to 1550 micro joules with a payload size of 1024 bytes. Figure 12 illustrates the performance of the proposed scheme over the existing security schemes PbMA and basic CoAP/DTLS when the payload size is varied from 64 to 1024 bytes. The proposed scheme reduces the header size and delay of communication due to the proper measurement of buffer size that considerably reduces the unnecessary packet loss. Moreover, Mixcolumns and S-Box increase used in the AES scheme of the existing payload-based authentication scheme increases the number of used registers and average energy consumption than the proposed scheme. For a payload size of 64 bytes, the proposed PEOS scheme spends 1170 micro joules, whereas the existing payload-based scheme PbMA and basic CoAP/DTLS spend 1220 and 1270 micro joules, respectively.

**Figure 13:**
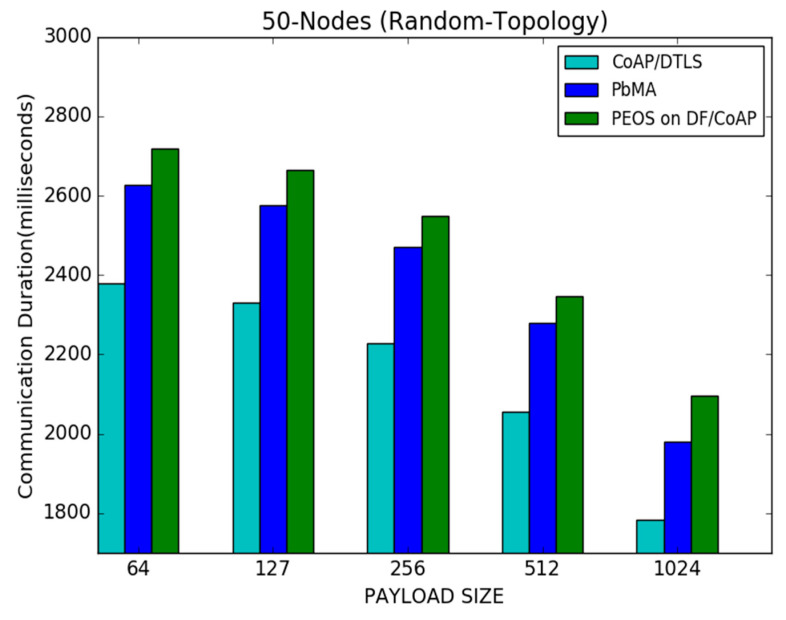
Payload size vs. communication duration for 50 node topology.

Figure 13 shows a communication duration performance for the PEOS, PbMA, and basic CoAP/DTLS schemes. It shows that the basic CoAP/DTLS outperforms both the existing payload-based authentication and the proposed scheme in all the scenarios. As with the payload size, improved performance in communication duration is achieved by all the schemes. The number of transmitted packets is reduced with a large payload size, and the communication duration is reduced compared to the scenario with a small payload size. For instance, with a payload size of 512 bytes, the proposed and existing payload-based encryption schemes, PEOS and PbMA [31], successfully transmit the data packets in 2300 milliseconds 2350 milliseconds, respectively. However, the basic CoAP/DTLS [8] delivers the packets in 2100 milliseconds. Moreover, identifying the exact buffer size and intimation of loss of exact fragments avoids the unnecessary packet loss and communication duration in the proposed scheme. For instance, in a payload size of 64 bytes, the proposed scheme PEOS delivers the packet in 2700 milliseconds. However, the existing payload-based scheme PbMA and basic CoAP/DTLS take 2620 s and 2390 milliseconds, respectively.

**Figure 14:**
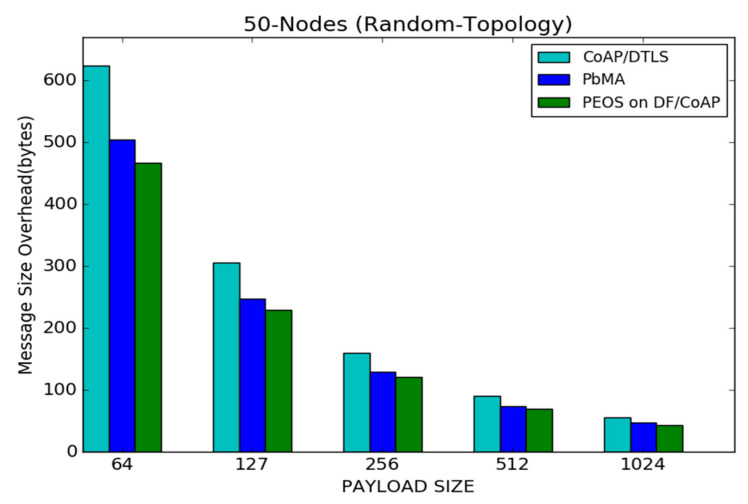
Payload size vs. message size overhead for 50 node topology.

Figure 14 shows the results of the message size overhead for proposed PEOS and existing PbMA and basic CoAP/DTLS schemes under various payload sizes. As observed, since the proposed scheme delivers data in a few packets with a large payload size, the message size overhead of all the schemes is considerable compared to the scenario of having a small payload size. For a payload size of 1024 bytes, the proposed authentication scheme experiences a message size overhead of 50 bytes, whereas the existing payload-based authentication scheme PbMA and basic CoAP/DTLS experience a message size overhead of 55 bytes and 65 bytes, respectively. In Figure 14, the proposed scheme experiences 230 bytes of message size overhead when the payload size is 127 bytes, whereas the message size overhead of the proposed scheme is reduced to 50 bytes with the payload size are maximum. For a payload size of 127 bytes, the CoAP/DTLS scheme experiences 310 bytes, whereas it is reduced to 65 bytes for a payload size of 1024 bytes. 

## 7. Conclusions

The proposed work has designed a mutual authentication scheme named Payload Encryption-based Optimisation Scheme for lightweight authentication (PEOS) on the Constrained Application Protocol (CoAP). Most of the existing works in CoAP security do not consider the overhead of Datagram Transport Layer Security (DTLS) in resource-constrained smart sensors. The proposed PEOS integrates and optimises some important features of DTLS in CoAP architecture to overcome this issue. It avoids the need to implement DTLS in a separate channel. Moreover, incorporating payload encryption and NACK messages improves the DTLS handshaking and re-transmission processes in PEOS. By removing the duplicate features of the protocol version and sequence number, the overhead is reduced significantly without impacting the performance of CoAP over distributed sensors. The proposed PEOS scheme exploits an Optimised Advanced Encryption Standard (OP-AES). Moreover, the PEOS implemented the parallel execution of S-boxes in SubBytes and delayed Mixcolumns in AES, and it successfully reduced the necessity of additional storage registers. Moreover, the PEOS implements the dynamic key generation process and avoids the chance of key traceability and guessing attacks in CoAP over distributed sensors. The utilization of token and dynamic key generation processes in PEOS avoids DDoS and confidentiality attacks. The efficiency of PEOS over distributed sensors in lightweight security and authentication is evaluated on Contiki OS using the Cooja simulator. Moreover, the proposed work is compared with the existing payload-based scheme and basic DTLS in the sensor network scenario. From the results, with the large payload size, the throughput of the proposed scheme is improved by 8.7% more than that of the existing PbMA by consuming 1550 microjoules under 50 nodes random topology.

## Figures and Tables

**Table 1:** Experimental parameters.

Parameters	Values
Application Layer	CoAP
Transport Layer	UDP
Network Layer	6LoWPAN
MAC Layer	IEEE 802.15.4
Total Number of Nodes	30 and 50
Simulation Area	100 m × 100 m
Transmission Range	50 m
Simulation Time	5 min
Payload Size	64,127,256,512,1024 bytes
Algorithms	CoAP/DTLS, PbMA, and PEOS on DF/CoAP

**Table 2:** Storage cost of the proposed PEOS and existing payload-based encryption scheme.

Type	Parameter	Proposed Work		Existing Work	
		Used in Smart Sensor	Used in Gateway	Used in Smart Sensor	Used in Gateway
Handshake Features	Nonce 1	Yes	No	Yes	No
	Nonce 2	No	Yes	No	Yes
	Token	Yes	Yes	Yes	Yes
AES Related Features	Secret Key	Yes	No	Yes	Yes
	Session key	Yes	No	Yes	Yes
	Seed value	Yes	No	No	No
	Potential Session Key	No	No	Yes	Yes
Total Storage Cost		640	384	640	640

## Data Availability

Not applicable.
